# Recurrent Kawasaki Disease in a Three-Year-Old Boy: A Case Report and Review of the Literature

**DOI:** 10.7759/cureus.98381

**Published:** 2025-12-03

**Authors:** Mohamad Sabsabee, Nur Sabsabee, Mira Elmiaari, Alia Magzoub, Farheen Khan, Moza Alhammadi, Maysa Saleh

**Affiliations:** 1 General Pediatrics, Al Jalila Children's Speciality Hospital, Dubai, ARE; 2 General Pediatrics, University of Kalamoon, Kalamoon, SYR; 3 Infectious Diseases, Al Jalila Children's Speciality Hospital, Dubai, ARE

**Keywords:** atypical kawasaki disease, coronary artery ectasia (cea), intravenous immunoglobulins (ivig), kawasaki disease (kd), recurrent kawasaki disease

## Abstract

Kawasaki disease (KD) is an acute medium-vessel vasculitis and the leading cause of acquired heart disease in children in developed countries. Recurrence is uncommon but clinically important because subsequent episodes may carry a heightened risk of coronary artery involvement and treatment resistance. We report a three-year-old boy with recurrent KD approximately 13 months after an initial, uncomplicated KD episode at 23 months of age. Echocardiography demonstrated coronary artery ectasia. He was diagnosed and treated with intravenous immunoglobulin (IVIG) on day 3 of illness and required steroid treatment in addition. Coronary artery ectasia has regressed on subsequent follow-ups. This case highlights that recurrent KD can present with variable clinical criteria yet demonstrate greater cardiac involvement and relative IVIG resistance compared with the index episode. Clinicians should maintain vigilance for recurrence, promptly institute anti-inflammatory therapy, and ensure close cardiology follow-up to mitigate coronary complications.

## Introduction

Kawasaki disease (KD), also known as mucocutaneous lymph node syndrome, is an acute systemic vasculitis that predominantly affects small- and medium-sized arteries, especially the coronary arteries. It is the leading cause of acquired heart disease in children in developed countries [1,2]. The etiology remains unclear, although it is believed to involve an abnormal immune response to infectious or environmental triggers in genetically susceptible hosts [3]. Diagnosis is clinical and based on the presence of prolonged fever (≥5 days) and at least four of the five principal features: bilateral non-purulent conjunctivitis, oral mucosal changes, polymorphous rash, peripheral extremity changes, and cervical lymphadenopathy [4]. Incomplete or atypical KD is diagnosed when fewer features are present but supported by laboratory or echocardiographic findings.

Timely treatment with high-dose intravenous immunoglobulin (IVIG) and aspirin significantly reduces the risk of coronary artery aneurysm (CAA) formation, which remains the most serious complication of KD [5]. Despite appropriate therapy, approximately 10-20% of patients demonstrate IVIG resistance, and 3-5% may develop coronary involvement despite treatment [6].

Recurrence of KD is defined as a repeat episode of Kawasaki disease occurring after complete resolution of the clinical signs and laboratory abnormalities from the initial episode. It is rare, reported in 2-4% of cases, with most recurrences occurring within two years of the first episode [7,8]. Recurrent cases tend to have more severe inflammatory features, a higher likelihood of IVIG resistance, and an increased risk of coronary artery abnormalities; Thus, corticosteroids and immunomodulators such as tumor necrosis factor-α inhibitors (e.g., infliximab) and interleukin-1 receptor antagonists (e.g., anakinra) play an important role in such cases [9]. Recognizing recurrence is challenging because symptoms may overlap with common childhood infections. Clinicians should remain vigilant, as delayed treatment in recurrent KD can lead to preventable cardiac complications.

We report a three-year-old boy who experienced a recurrent episode of KD approximately one year after his initial illness. The recurrence was characterized by coronary ectasia - dilatation of an artery that does not meet the threshold for aneurysm - and IVIG resistance, emphasizing the importance of prompt recognition and ongoing cardiologic follow-up in children with a prior history of KD.

## Case presentation

A three-year-old Arab boy presented to the emergency department with a three-day history of high-grade fever (T max 40 °C) and a diffuse erythematous maculopapular rash, initially appearing on the trunk and back before spreading to the face and extremities. He also had a one-day history of non-purulent conjunctivitis, dry, cracked lips, and diarrhea. On examination, he appeared unwell and mildly dehydrated, with hepatomegaly (4 cm below the costal margin) and bilateral, non-tender cervical lymphadenopathy (measuring 2×3 cm). No changes in the extremities were noted.

Initial laboratory investigations, summarized in Table 1, demonstrated leukocytosis with neutrophilia and lymphopenia along with microcytic hypochromic anemia. Inflammatory markers (C-reactive protein (CRP), procalcitonin, and erythrocyte sedimentation rate (ESR)) were markedly elevated. In addition, ferritin, D-dimer, and N-terminal pro-B-type natriuretic peptide (NT-pro-BNP) were increased.

**Table 1:** Laboratory test results WBC: white blood cells, ESR: erythrocyte sedimentation rate, CRP: C-reactive protein, NT-proBNP: N-terminal pro-B-type natriuretic peptide, ALT: alanine transferase

Parameter	Result	Normal range
Hemoglobin	10.1 g/L	11.0–14.0 g/L
WBC count	15.1 × 10^3^/µL	4–12 × 10^3^/µL
Neutrophils	13.3 × 10^3^/µL	1.5–8.0 × 10^3^/µL
Lymphocytes	1.2 × 10^3^/µL	2.0–8.0 × 10^3^/µL
Platelets	325 × 10^3^/µL	150–400 × 10^3^/µL
ESR	60 mm/hr	<20 mm/hr
CRP	91.5 mg/L	0-5 mg/L
Procalcitonin	7.13 ng/mL	<0.5 ng/mL
Ferritin	236 ng/mL	5.3-99.9 ng/mL
Fibrinogen	684 mg/dL	216-401 mg/dL
D-dimer	5.44 µg/mL	<0.5 µg/mL
NT-proBNP	1967 pg/mL	<125 pg/mL
ALT	189 U/L	0-39 U/L
Albumin	2.6 g/dL	3.8–5.4 g/dL

Given the constellation of mucocutaneous findings, elevated inflammatory markers, and cardiac enzyme elevation, a diagnosis of recurrent KD was established on day 3 of the illness. However, he was started on ceftriaxone empirically, which was discontinued once blood and urine cultures came back sterile. In addition, viral serologies for *Mycoplasma pneumoniae*, Epstein-Barr virus, measles, Rubella, and severe acute respiratory syndrome coronavirus 2 (SARS‑CoV‑2) were negative, along with a normal antistreptolysin O level.

He had a prior history of typical KD at 23 months of age, when he presented with 5 days of fever, maculopapular rash, strawberry tongue, non-purulent conjunctivitis, and cervical lymphadenopathy. Laboratory findings at that time showed the elevation of inflammatory markers to a lesser extent (CRP: 45 mg/L, ferritin: 100 ng/mL ), and he responded well to IVIG (2 g/kg) administered on day 5 of illness. Echocardiography at that time revealed normal coronary arteries without valvular involvement, and follow-up imaging remained normal.

**Figure 1:**
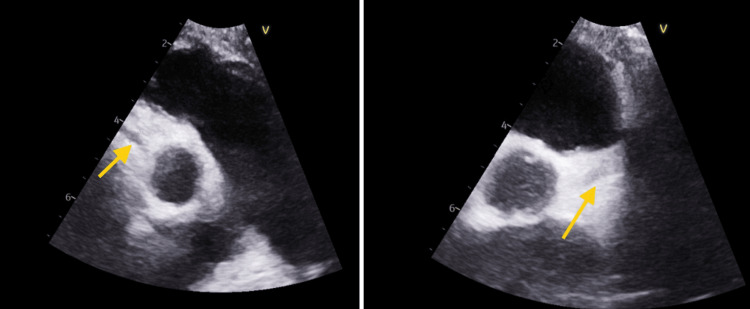
Echocardiography at presentation Left arrow: RCA 2.4 mm; Right arrow: LMCA 3.3 mm RCA: right coronary artery; LMCA: left main coronary artery

At the current presentation, echocardiography showed moderate mitral and tricuspid regurgitation with coronary ectasia (left anterior descending artery (LAD) 3.3 mm, z + 4.5; left main coronary artery (LMCA) 3.2 mm; right coronary artery (RCA) 2.4 mm) as seen in Figure 1. The patient was treated with IVIG (2 g/kg single infusion) and high-dose aspirin (80 mg/kg/day). Repeat echocardiography on day 3 of admission revealed partial regression of coronary dilation (LAD 2.7 mm, z + 2.2) with persistent distal uniformity. Despite appropriate therapy, the fever persisted beyond 48 hours, prompting escalation to IV methylprednisolone (10 mg/kg/day for three days). The fever subsequently resolved, and the patient’s overall condition improved markedly. Repeat laboratory results before discharge demonstrated normalization of inflammatory markers (CRP 3 mg/L, procalcitonin 0.1 ng/mL, ESR 30 mm/hr).

**Figure 2:**
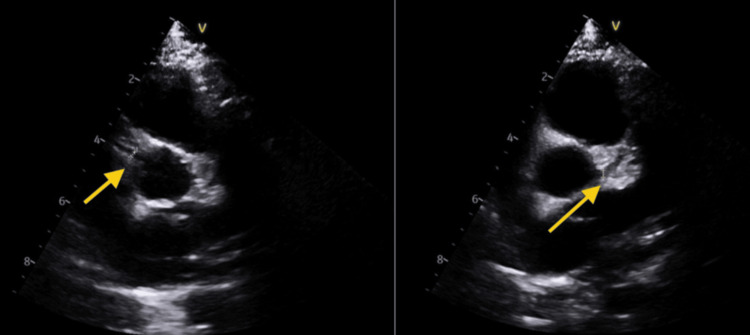
Echocardiography at the two-week follow-up Left arrow: RCA 2 mm; Right arrow: LMCA 2.2 mm RCA: right coronary artery; LMCA: left main coronary artery

Two weeks later, follow-up echo revealed normal coronary dimensions (LAD 2.0 mm; LMCA 2.2 mm; RCA 2.0 mm) as seen in Figure 2. He was maintained on low-dose aspirin for eight weeks, which was discontinued after confirming complete normalization of echocardiographic findings, including mitral regurgitation. The child remained asymptomatic at subsequent follow-ups and returned to his baseline health.

## Discussion

Recurrent KD is relatively rare, occurring in approximately 2-4% of affected children, with most recurrences developing within two years of the initial episode [1,2]. The underlying mechanisms remain unclear but are thought to involve persistent immune dysregulation, genetic predisposition, or incomplete resolution of vascular inflammation from the initial illness [3].

Risk factors for recurrence

Similar to our patient, younger age at disease onset (typically under three years), male sex, and specific genetic polymorphisms, particularly in ITPKC, CASP3, and HLA class II genes, have been associated with increased recurrence risk [3,4]. These genes regulate T-cell activation and apoptosis, and their variants are believed to promote prolonged or exaggerated inflammatory responses. Our patient, a three-year-old male, falls within this high-risk demographic. These findings and other treatment implications are summarized in Table 2.

**Table 2:** Summary of treatment insights from previous articles KD: Kawasaki disease; IVIG: intravenous immunoglobulin; CAA: coronary artery aneurysm

Study / Author (Year)	Study Design / Region	Population & Sample Size	Focus / Main Findings	Coronary or Recurrence Data	Treatment Insights
Nakamura et al., 1994 [1]	Nationwide survey, Japan	3,000 + KD cases	First national analysis of KD recurrence	Recurrence ≈ 3.5%; most within 2 years	Early IVIG → lower CAA risk
Hirata et al., 2001 [2]	Follow-up nationwide survey, Japan	16,000 KD cases	Identified risk factors for recurrence	Recurrence 2.9%; higher in males < 3 years	Younger age, male sex → higher recurrence risk; highlights early follow-up necessity
Burns et al, 2004 [3]	Review	—	Overview of KD pathogenesis and immune basis	Recurrent KD linked to immune dysregulation	Prompt IVIG within 10 days → ↓CAA
Kim et al., 2011 [4]	Genetic association study, Korea	391 patients + controls	Identified ITPKC, CASP3, HLA variants	Genetic polymorphisms ↑ recurrence risk	Genetic screening may guide prognosis
Ha et al., 2013 [5]	Meta-analysis	13 studies, 2,538 cases	Incomplete KD → higher coronary risk	CAA in 25% of incomplete vs 10% complete KD	Highlights diagnostic vigilance
Hsieh et al., 2002 [6]	Observational, Taiwan	56 children	Clinical profile of atypical KD	Atypical cases → ↑ diagnostic delay → ↑ CAA rate (18%)	Early IVIG recommended, despite incomplete criteria
Holman et al., 2010 [7]	National hospitalization database, USA (1997–2007)	19,000 hospitalizations	Trends in KD hospitalization and mortality	Cardiac complications ~12%; recurrence ~2%	Importance of early IVIG treatment
Dionne et al., 2018 [8]	Review	—	Myocarditis as early manifestation of KD	Found myocarditis common (up to 70%) early in KD	Early steroids beneficial in IVIG-resistant KD
McCrindle et al., 2017 [9]	AHA scientific statement	Expert consensus	Updated global guideline for KD management	IVIG ± steroids reduce CAA to ~3–5%	Standardized steroid protocols improve outcome
Choi et al., 2010 [10]	Case report	1 child with KD + abscess mimic	KD can mimic deep-neck infection	Diagnostic delay increased CAA risk	Emphasized differential diagnosis awareness

Clinical comparison between index and recurrent episodes

Published data show that 70-80% of initial KD episodes present with complete diagnostic criteria, while only 40-50% of recurrences do so, with the remainder exhibiting incomplete or atypical forms [5,6]. Cardiac involvement is reported in 8-10% of first-time cases but rises to 20-30% with recurrence, and IVIG resistance increases from 10-20% initially to nearly 30% in subsequent episodes [7,8].

Our patient’s second episode was associated with significant coronary artery ectasia (LAD 3.3 mm, z-score +4.5) and moderate mitral and tricuspid regurgitation, despite treatment being initiated on day 3 of fever, earlier than the usual day 5-7 window. This indicates that recurrence can follow a more aggressive inflammatory course even with prompt therapy.

Atypical presentations

Recognizing recurrent or incomplete KD can be challenging because its features often overlap with infectious diseases. Cases have been misdiagnosed as retropharyngeal or parapharyngeal abscesses, toxic shock syndrome, or viral exanthems, leading to treatment delays and increased cardiac complications [9,10]. Such mimics are particularly problematic in incomplete or recurrent KD, where classical mucocutaneous findings may be subtle or absent. Awareness of these atypical patterns and maintaining a high index of suspicion in febrile children, especially those with a prior KD history, is essential.

Management and outcomes

Our patient required adjunctive corticosteroid therapy after failing to defervesce (typically defined as persistent or recrudescent fever ≥36 hours after infusion) with IVIG alone. The addition of methylprednisolone resulted in rapid clinical improvement and normalization of inflammatory markers. Similar to previously reported experiences, corticosteroids have proven effective in IVIG-resistant KD, reducing fever duration and possibly mitigating coronary complications without increasing adverse events [9]. Serial echocardiograms in our case demonstrated complete resolution of coronary ectasia and valvular regurgitation within one month, reinforcing the importance of early recognition and combined therapy.

Corticosteroids are now increasingly recognized as effective adjuncts in IVIG-resistant KD, reducing fever duration and CAA risk, with efficacy rates reported at 70-80% when used as second-line therapy [10]. Potential adverse effects-transient hypertension, hyperglycemia, and electrolyte imbalance-are typically mild and reversible with short-course regimens.

Clinical implications

Recognition of recurrent KD can be challenging, as clinicians may initially attribute fever and rash to infectious etiologies, especially if the prior episode was remote. Given that recurrent KD may exhibit either complete or incomplete features but with greater inflammatory intensity, echocardiography should be performed early in any suspected case, and IVIG should not be delayed even if fever duration is under five days when clinical suspicion is high. Continuous cardiology follow-up is essential, as recurrent inflammation may predispose to late-onset coronary remodeling despite apparent recovery [10].

## Conclusions

Recurrent Kawasaki disease (KD) is uncommon but may present with greater inflammatory severity and higher risk of cardiac complications than the initial episode, even when recognized and treated early. Clinicians should maintain a high index of suspicion for recurrence in any febrile child with prior KD, as incomplete or atypical presentations can mimic infectious illnesses and delay therapy. Early echocardiography, prompt IVIG administration, even before five days of fever, and timely escalation in IVIG-resistant cases are essential to prevent coronary sequelae and ensure full cardiac recovery.
